# Fatigue and cognition: Pupillary responses to problem‐solving in early multiple sclerosis patients

**DOI:** 10.1002/brb3.717

**Published:** 2017-05-17

**Authors:** Sigrid A. de Rodez Benavent, Gro O. Nygaard, Hanne F. Harbo, Siren Tønnesen, Piotr Sowa, Nils I. Landrø, Marte Wendel‐Haga, Lars Etholm, Kristian B. Nilsen, Liv Drolsum, Emilia Kerty, Elisabeth G. Celius, Bruno Laeng

**Affiliations:** ^1^ Department of Ophthalmology Oslo University Hospital and Institute of Clinical Medicine University of Oslo Oslo Norway; ^2^ Department of Neurology Oslo University Hospital and Institute of Clinical Medicine University of Oslo Oslo Norway; ^3^ Department of Psychology University of Oslo Oslo Norway; ^4^ Department of Radiology Oslo University Hospital and Institute of Clinical Medicine University of Oslo Oslo Norway; ^5^ Department of Neurophysiology Oslo University Hospital Oslo Norway; ^6^ Department of Neuroscience Norwegian University of Science and Technology Trondheim Norway; ^7^ Department of Neurology Oslo University Hospital Oslo Norway

**Keywords:** cognition, fatigue, multiple sclerosis, pupillometry

## Abstract

**Introduction:**

In early multiple sclerosis (MS) patients, cognitive changes and fatigue are frequent and troublesome symptoms, probably related to both structural and functional brain changes. Whether there is a common cause of these symptoms in MS is unknown. In theory, an altered regulation of central neuropeptides can lead to changes in regulation of autonomic function, cognitive difficulties, and fatigue. Direct measurements of central neuropeptides are difficult to perform, but measurements of the eye pupil can be used as a reliable proxy of function.

**Methods:**

This study assesses pupil size during problem‐solving in early MS patients versus controls. A difference in pupil size to a cognitive challenge could signal altered activity within the autonomic system because of early functional brain changes associated with cognitive load. We recruited MS patients (mean disease duration: 2.6 years, *N *= 41) and age‐matched healthy controls (*N *= 43) without eye pathology. Neurological impairment, magnetic resonance imaging, visual evoked potentials, depression, and fatigue were assessed in all of the patients. In both groups, we assessed processing speed and retinal imaging. Pupil size was recorded with an eye‐tracker during playback of multiplication tasks.

**Results:**

Both groups performed well on the cognitive test. The groups showed similar pupillary responses with a mean of 0.55 mm dilation in patients and 0.54 mm dilation in controls for all the tasks collapsed together. However, controls (*N *= 9) with low cognitive scores (LCS) had an increased pupillary response to cognitive tasks, whereas LCS MS patients (*N *= 6) did not (*p *< .05). There was a tendency toward a smaller pupillary response in patients with fatigue.

**Conclusions:**

This is the first study to investigate pupillary responses to cognitive tasks in MS patients. Our results suggest that MS‐related changes in cognition and fatigue may be associated with changes in arousal and the autonomic regulation of task‐related pupillary responses. This supports the theory of a link between cognition and fatigue in MS.

## 1 INTRODUCTION

Multiple sclerosis (MS) patients frequently suffer from cognitive difficulties and fatigue, often present early in the disease course (Amato, Ponziani, & Pracucci, 1995; Krupp, Alvarez, LaRocca, & Scheinberg, 1988). The impact on everyday life may be severe (Nortvedt, Riise, Myhr, & Nyland, 1999). Neuropsychological testing, functional and structural brain imaging are now extensively used to investigate cognition (Amato et al., 2010; Bakshi, 2003). However, the cognitive symptoms are only partly explained by changes in the central nervous system (CNS) as demonstrated by magnetic resonance imaging (MRI) studies (Genova et al., 2013; Rocca, Parisi, et al., 2014; Rocca et al., 2015). Early brain changes in MS may affect central neurotransmitters and the autonomic nervous system, which could be effectively picked up with alternative methods that relate directly to autonomic function like pupillometry. Moreover, the possibility to measure this through the eye pupil is less expensive than MRI methods and with currently infrared eye‐tracking systems is also elegantly noninvasive (Joshi, Li, Kalwani, & Gold, 2016). To the best of our knowledge, investigations of task‐evoked pupillary responses have not been applied yet to this patient group despite the relationship between such a physiological measurement and cognitive effort. In this study, we therefore explored whether cognitive challenges and fatigue in early MS would be reflected in these patients’ pupillary responses to problem‐solving tasks.

Functional MRI (fMRI) studies have indicated that patients with MS or clinically isolated syndromes (CIS) show a different cerebral ‘resting state’ activation as well as task‐related cerebral activation patterns compared to healthy controls (Audoin et al., 2003; Forn et al., 2006; Penner, Rausch, Kappos, Opwis, & Radü, 2003; Rocca, Parisi, et al., 2014; Roosendaal et al., 2010; Staffen et al., 2002). These altered cerebral activation patterns in MS patients may be signs of functional reorganization compensating for structural damage. Alternatively, such a reorganization may be dysfunctional and contribute to a less appropriate brain activation, cognitive difficulties, and fatigue.

fMRI studies of fatigue in MS are scarce, but some studies have found both altered resting state and different activation patterns in fatigued MS patients compared to healthy controls (DeLuca, Genova, Hillary, & Wylie, 2008; Engström, Flensner, Landtblom, Ek, & Karlsson, 2013; Genova et al., 2013). An intriguing possibility is that connectivity in partially‐overlapping networks collapses over time and structural damage may lead to fatigue and cognitive difficulties (Hanken, Eling, & Hildebrandt, 2014). This hypothesis is supported by the observation that patterns of gray matter atrophy are similar in patients with fatigue and cognitive difficulties in MS (Calabrese et al., 2010; Pellicano et al., 2010), in contrast to the weak correlation between these symptoms and damages to normal‐appearing white matter and white matter lesion load. A thalamo‐striato‐frontal disruption pattern has been suggested as the cause of MS‐related fatigue (Pardini, Bonzano, Mancardi, & Roccatagliata, 2010). A similar disconnection syndrome has been proposed to explain MS‐related cognitive difficulties (Louapre et al., 2014). Such a disconnection may not only be structural; a possible cause of the altered cerebral activation patterns in patients with cognitive difficulties and fatigue could be because of disturbances in regulatory neuropeptides. The role of noradrenergic activation of CNS is particularly interesting in relation to these symptoms as noradrenergic activation leads to wakefulness and is involved in the allocation of attention (Laeng, Sirois, & Gredeback, 2012).

Noradrenergic activation can be observed indirectly by measuring pupil size during task‐related pupillary responses (Joshi et al., 2016; Szabadi, 2013). The pupil size in general depends on arousal, sleepiness and sleep‐deprivation, demands on attention, and surrounding light conditions as well as visual acuity and refractional errors. It is governed by the autonomic nervous system, and alterations in the sympathetic or parasympathetic nervous system possibly affects the task‐evoked pupillary response as well.

Pupillometry has been used in psychological research as a marker of intensity of mental activity and of changes in mental states. The task‐evoked pupillary response provides a reliable and sensitive indicator of within‐patient variations in processing load in memory, language, reasoning and perception tasks, and it is sensitive to between‐group differences in intelligence (Beatty, 1982). At the neural level, this pupillary response is associated with the activation of locus coeruleus, a brainstem nucleus and hub of the noradrenergic system, that is involved in arousal and the allocation of attention (Alnæs, Sneve, Espeseth, Pieter, & Laeng, 2014; Murphy, O'Connell, O'Sullivan, Robertson, & Balsters, 2014). Both the parasympathetic inhibition and the sympathetic activation of pupillary dilator muscle are involved in regulating the pupillary response to cognitive tasks (Steinhauer, Siegle, Condray, & Pless, 2004). Studying the pupillary dilation may therefore be a suitable noninvasive technique to examine deficits of the catecholamines’ and cholinergic system as well as the autonomic nervous system in general. More specifically, pupillary studies can give insight into deficits of cognition resulting from neurological disorders.

Optic neuritis (ON) is common in patients with MS and can possibly interfere with pupillary measurements. It constitutes the first symptom of the disease for 25% of MS patients and occurs in the course of the disease in about 70% (Toosy, Mason, & Miller, 2014). It is not known whether the disruption of the visual pathways caused by ON leads to an altered task‐related pupillary response in MS patients. Ophthalmologic assessment and evaluation of visual evoked potentials (VEP) should therefore prelude the application of this method in MS patients. Further, lesions of the brainstem could lead to a disruption of the tracts from LC and other brainstem nuclei and interfere with the regulation of the pupil size. MRIs of the brainstem should therefore be evaluated neuroradiologically in MS patients subject to this method. In this study, we combined a multidisciplinary approach with examinations of task‐related pupillary responses. A history of optic neuritis or brainstem lesions could then be tested for interference with the pupillary responses in a group of early MS patients, that is, within a time from diagnosis of less than 4 years.

Our main hypothesis was that pupillary responses to problem‐solving in early MS patients would be different from those of healthy controls. One possible prediction was that neurodegeneration in connectivity could reduce the neuromodulatory influence of the norepinephrine —Locus Coeruleus system, resulting in reduced pupillary dilations during cognitive challenges task (Granholm, Morris, Sarkin, Asarnow, & Jeste, 1997). In addition, we examined whether pupillary responses to problem‐solving would be influenced by individual differences in cognition, depressive symptoms, and fatigue, and in the subgroups with and without ON and brainstem lesions. As we had no a priori knowledge of the direction of the possible group differences, we used an exploratory approach with two‐tailed statistical testing.

## 2 MATERIALS AND METHODS

### 2.1 Patients and controls

Relapsing‐remitting multiple sclerosis (RR MS) patients (*n *= 49) diagnosed according to the revised McDonald Criteria (Polman et al., 2011), initially enrolled in a study of cognition and neuroimaging (*n *= 76) (Nygaard et al., 2015), were asked to participate in this study. Healthy controls were recruited from the local community, the hospital and university environment by email or direct inquiry. Inclusion criteria for both groups were—age 18–50 years, fluency in Norwegian, no prior ophthalmological, neurological or psychiatric disease, no head injury, and no substance abuse. To avoid possible confounding by reduced visual acuity, refractive errors with a spherical equivalent of more than ±6 or lesions of the visual pathways were exclusion criteria. All participants underwent an ophthalmological examination. The MS patients were also examined with visual evoked potentials (VEP). We could therefore carefully select measurements from the healthy eyes of the participants. Eight patients were excluded [due to bilateral ON (*n *= 5), previous amotio retina (*n *= 1), other conditions that may have interfered with the measurements (*n *= 2)], leaving 41 patients eligible for analysis. Forty‐seven healthy controls were considered for the experiment, of whom four were excluded [due to technical difficulties with the eye‐tracker (*n *= 2) and other medical or neurological conditions (*n *= 2)]; thus, 43 controls were eligible for analyses. The patients were clinically stable with at least 3 months since an episode of ON in the contralateral eye and 6 weeks since any other relapse or corticoid treatment.

All participants gave written informed consent and the study was approved by the regional ethical committee of South Eastern Norway (REK).

### 2.2 Neurological and neuropsychological examinations

The patients underwent a full neurological examination within 2 weeks of the ophthalmological and pupillary measurements. The Expanded Disability Status Score (EDSS) was used to assess neurological disability. Depressive symptoms were assessed with the self‐report Beck Depression Inventory (Beck, Steer, & Brown, 1996) addressing both cognitive and somatic aspects of depression, and fatigue was reported with the self‐report Fatigue Severity Scale (FSS) (Krupp, LaRocca, Muir‐Nash, & Steinberg, 1989) to separate depression from fatigue. Patients with FSS >4 were considered to have fatigue and patients with BDI >12 were considered to have depression.

Both patients and controls underwent testing with the neuropsychological tests included in the test battery “Brief International Cognitive Assessment for Multiple Sclerosis” (BICAMS) (Langdon et al., 2011). Symbol Digit Modalities Test (SDMT) (Smith, 1982) was used to assess processing speed, the sum of the first five trials of the California Verbal Learning Test 2 (CVLT) (Delis, Kramer, Kaplan, & Ober, 2000) was used to test verbal memory and the sum of the first three trials of the Brief Visuospatial Memory Test‐Revised (BVMT) (Benedict, 1997) was used to test visuospatial memory. The raw scores of the test results of the controls were used to create z‐scores for the patients. We chose a cut‐off of z < −2 for CVLT and BVMT and a cut‐off for SDMT results of z < −1.5. Anyone who scored z < −2 on either BVMT or CVLT, or z < −1.5 on SDMT were classified as with a low cognitive score (LCS). The rest were classified as with a normal cognitive score (NCS).

### 2.3 Ophthalmological and pupillometric examinations

All participants underwent an ophthalmological examination, including the swinging flashlight test, as well as visual acuity measured as the logarithm of the minimum angle of resolution (logMAR) and spherical assessment. Pupil data were acquired using the SMI (SensoMotoric Instruments, Teltow, Germany) R.E.D. eye‐tracking device. All participants were seated approximately 70 cm from the monitor in the same room, lit with approximately 180 lux. The eye pupillary responses were registered with I‐View Software (SMI). The pupil diameter of both eyes was measured at a sampling rate of 60 Hz. The RED can operate at a distance of 0.5–1.5 m. This device has two sources of infrared light from an infrared light‐sensitive video camera, placed under the monitor frame. The RED keeps track of head position which allows measuring reliably the pupil diameters in mm, despite the presence of head movements. According to SMI specifications, the RED system can detect changes as small as 0.004 mm. Binocular data were recorded at a sampling rate of 60 Hz (i.e., every 16 ms). The constant display resolution was set to 1680 × 1050 pixels. A 5‐point calibration pattern was displayed to participants before running the eye‐tracker sessions. A dispersion of <0.5 in both x‐ and z‐space was considered a successful calibration; recalibration was initiated until a successful calibration was obtained. Measurements were randomly conducted throughout the day for both groups. Patients and controls were examined during the same period, but the examiners were not blinded regarding the status of the participants.

### 2.4 Optic coherence tomography

Retinal imaging was performed by the same trained ophthalmologist (SADRB) with the spectral domain RS‐3000 OCT Retina Scan (Nidek Inc., CA, USA). Peripapillary retinal nerve fiber layer thickness (RNFL) data were obtained with the Disc Circle protocol with a scan width of 3.45 mm and a scanning speed of 53,000 A‐scans/sec, centered on the optic nerve head without crossing of the two inner scan circles. All scans included had a signal strength of 8/10 or better.

### 2.5 Visual evoked potentials

Visual evoked potentials (VEP) delay to P100 were obtained with dimmed light (~25 lux) and the screen placed 100 cm from the eyes of the patients, with a Dantec Keypoint Focus system with checkerboard patterns (check size 65’) presented at 2 Hz with a 16” cathode ray tube screen. Three hundred responses were averaged from the mid‐occipital lobe (MO, defined to be 5 cm above inion) to the mid‐frontal lobe (Fz, as defined by the 10/20 system) with 1 Hz – 100 Hz band‐pass filter. Rejection level was set to ± 100 *μ*V. The VEP results were evaluated by two experienced clinical neurophysiologists (KBN and LE), and VEP was regarded as pathological with a duration of more than 110 ms and/or with a greater delay of at least 6 ms compared to the contralateral eye.

### 2.6 Magnetic resonance imaging

All patients underwent cerebral MRI examinations using the same 1.5 T Siemens Avanto scanner (Siemens Medical Solutions) with a 12‐channel head coil. For clinical radiological evaluation, FLAIR, T2 and pre‐ and post‐gadolinium T1 MP‐RAGE sequences were used. Details concerning the sequences have been described earlier (Nygaard et al., 2015). For this study, we were interested in detecting the presence of brainstem lesions. An experienced neuroradiologist (PS), blinded to the pupillometry and clinical test results, evaluated the MRIs and rated the patients as with or without lesions of the brainstem.

### 2.7 Experimental design

**Figure 1:**
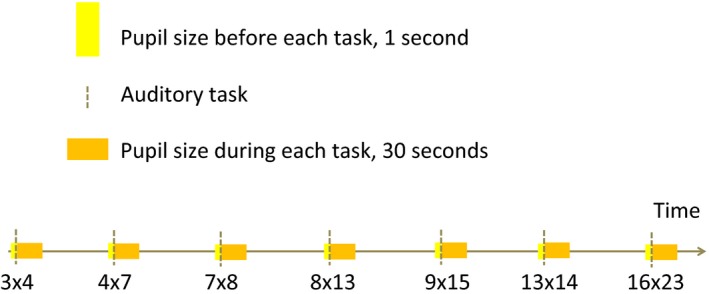
Experimental design. The participants were presented auditorily with mathematical tasks of increasing difficulty while they were told to fixate on a cross on a gray computer screen. The pupil size was measured continuously before and after the oral responses to the tasks

New infrared eye‐tracker technology was employed to reproduce a classic pupillometric experiment of cognitive load on patients and controls. Playback of seven mathematical multiplication tasks of increasing difficulty, adapted from the classic study of Hess and Polt (Hess & Polt, 1964) were presented auditorily at the initiation time (Figure 1). Continuous pupil size recordings for each task started one second before the initiation time and lasted for 30 s after, irrespective of the participants’ oral response time. The oral response time was recorded by a tap on the keyboard by a trained research assistant, coregistering whether the response was correct.

#### 2.7.1 Statistical analysis of baseline characteristics and neuropsychological test results

SPSS Statistics version 22.0 (SPSS, Chicago, IL, USA) was used for statistical analyses. Descriptive statistics of the differences between patients and controls were performed using independent samples *t* tests for continuous data and χ^2‐^ tests for categorical data. Bonferroni‐corrections for multiple comparisons were applied when appropriate. Pearsons correlations were used to test for association between the peak of the pupillary response and possible confounders.

#### 2.7.2 Statistical analyses of pupillary response to the experimental task

Initiation time and oral response of each task were coregistered with the dynamic measurements of the pupil size. The mean pupil size, during and after the participants’ oral responses were extracted, using the open source programming language C++ (http://www.cprogramming.com/). The data were then transferred to SPSS for statistical analyses.

The pupillary dilation was calculated as percent difference in pupil size from each pre‐task baseline: [(mean pupil size of each second during the task/task‐dependent baseline pupil size) −1] × 100. The results were analyzed using repeated measure analyses of variance (ANOVA), with group (e.g., patients or controls) as independent variables, and pupillary dilation during the task as dependent variables. To control for possible confounders, we performed relevant analyses with analyses of covariance (ANCOVA), with age, gender, and brainstem lesions as cofactors. For the ANOVA and ANCOVA analyses, we tested for differences in the dependent variables throughout the experiment (main effect), for differences in the dependent variable between patients and controls (between‐group effect), and for an interaction between the two (interaction effect).

To assure that any group differences were not caused by the lack of a pupillary response of participants not responding or by error‐related negativity (i.e., increased cognitive processing after making a mistake), we performed relevant analyses in subgroups of participants with trials with only correct answers.

A significance level of *p *< .05 was applied to all analyses.

## 3 RESULTS

### 3.1 Clinical characteristics of patients and controls

The demographic characteristics of patients and controls are summarized in Table 1. The groups were matched on age (*t* = 1.12, *p *= .222) and gender (χ^2^ = 0.0214, *p *= .884). The controls had on average an education level 2 years higher than the patients (*t* = −3.31, *p *= .001).

**Table 1:** Baseline characteristics

	Patients	Controls
	*n *= 41	*n *= 43
Gender, female, *n* (%)	28 (68)	30 (70)
Age, years, mean (*SD*)	35 (7.4)	33 (6.7)
Education, years, mean (*SD*)	15 (2.1)	17 (3.0)^a^
Disease duration, years, mean (*SD*)	2.6 (2.1)	–
Time since diagnosis, years, mean (*SD*)	1.6 (0.9)	–
Neurological disability, EDSS, mean (*SD*)	1.9 (0.8)	–
Depressive symptoms, BDI, mean (*SD*)^b^	7 (5.9)	–
Fatigue, FSS, mean (*SD*)^b^	4 (1.7)	–
Brain stem lesions on MRI, *n* (%)^c^	22 (69)	–
Disease modifying treatment		
None, *n* (%)	7 (17)	–
First line, *n* (%)	30 (73)	–
Second line, *n* (%)	4 (10)	–

a Difference between patients and controls, *p *= .001.

b Data available on 40 patients.

c Data available on 32 patients.

### 3.2 Neurological and neuropsychological test results

All patients had a mean disease duration of less than 4 years and a low disability level with a mean EDSS of 1.9 and a median EDSS of 1.5. MRI revealed that 69% of the patients had brainstem lesions, and the presence of brainstem lesions were thus included as covariate in analyses of the experimental task results.

The neuropsychological test results of the patients have previously been reported, comparing their results to published norms (Nygaard et al., 2015). The controls performed well on SDMT (mean 56.1, *SD* 8.1, median 56) and BVMT (mean 27.4, *SD* 4.9, median 29). They had very high scores on CVLT (mean 70.5, *SD* 6.3, median 72); most of the controls were able to list all 16 words of the word list on the second trial. The patients performed on average worse than the healthy controls on the CVLT, and similarly on the BVMT and SDMT. Similar proportions of patients and controls failed at least one neuropsychological test (LCS: 9/39 patients and 6/41 controls, χ^2^ = 0.463, *p *= .496). Two patients failed two of the neuropsychological tests, whereas none failed three tests. Z‐scores of the neuropsychological tests were generated from the raw scores of the controls. Both CVLT and BVMT results of the controls showed a right hand skewness (CVLT skewness: −0.9, BVMT skewness: −0.642), while SDMT results of the controls were normally distributed.

As previously reported (Nygaard et al., 2015), the patients had a mean FSS of 4 (*SD* 1.7), and 48% had a mean FSS >4 and were thus classified as with fatigue. The mean BDI was 7 (*SD* 5.9) and 25% were classified as with depressive symptoms with BDI >12.

#### 3.2.1 Ophthalmological test results of patients and controls

The patients and controls had similar visual acuity and spherical equivalents on both the tested and the contralateral (non‐tested) eye. The patients had a thinner RNFL than the controls in the tested eye, and even thinner RNFL on the contralateral (non‐tested) eye, where 51% of the patients had a history of ON. No relative afferent pupillary defect was observed on the tested eyes (Table 2).

**Table 2:** Results of the eye examinations

	Patients	Controls
	*n *= 41	*n *= 43
History of optic neuritis on any eye, *n* (%)	21 (51)^a^	0 (0)
Left eye tested with pupillometry, *n* (%)	32 (78)^a^	42 (98)
Visual acuity of tested eye, Log MAR, mean (*SD*)	−0.05 (0.10)	−0.08 (0.07)
Visual acuity of other (non‐tested) eye, Log MAR, mean (*SD*)	−0.05 (0.11)	−0.06 (0.11)
Spherical equivalent of tested eye, mean (*SD*)	−0.67 (1.74)	−0.58 (1.35)
Spherical equivalent of other (non‐tested) eye, mean (*SD*)	−0.73 ± 1.40	−0.61 (1.83)
Retinal nerve fiber layer thickness of tested eye, mean (*SD*)	98.9 ± 10.2^a^	104.6 (11.3)
Retinal nerve fiber layer thickness of other (non‐tested) eye, mean (*SD*)	94.8 ± 13.9^a^	104.6 (11.5)
VEP of tested eye^b^, Latency to p100, mean (*SD*)	104.7 (5.0)	–
VEP of other (non‐tested) eye^b^, Latency to p100, mean (*SD*)	108.7 (7.0)	–

Approximately, half of the patients had a history of optic neuritis. In the patients with a history of optic neuritis on one eye, data from the other eye were used in the analyses. Visual evoked potentials (VEPs) were only tested in the patients and were longer in the non‐tested than in the tested eyes, probably because of a history of optic neuritis in a large proportion of the non‐tested eyes.

a Difference between patients and controls, *p *< .05.

b Test results of 40 patients. For one patient neurophysiological visual evoked potential test results were inconclusive.

For the pupillometric analyses, we used the results from the left eye of the participants, except from the participants with a history of left eye ON, prolonged VEP latency (eight patients) or other pathology (one patient and one control with left side amblyopia). In these cases, the results from the right eye were used.

### 3.3 Task results of the experiment

**Figure 2:**
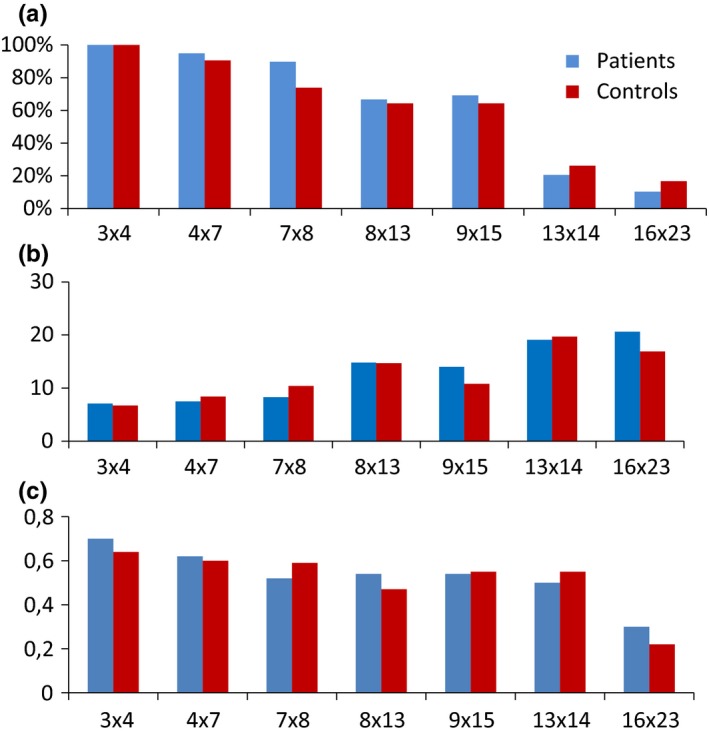
Experimental test results. (a) The percent of participants with correct answers, (b) the time spent to answer (seconds), and (c) pupillary dilation (millimeter) for each mathematical task is illustrated. Patients and controls had similar proportions of correct answers, spent similar time to complete the mathematical tasks and had similar pupillary dilations for each task

The patients and controls performed comparably well on the arithmetic calculations [patients mean 4.5 (*SD* 1.2) correct responses, controls mean 4.4 (*SD* 1.6) correct responses]. All the participants gave a correct reply to the easiest task, while only a few participants gave correct replies to the most demanding tasks. The patients and controls had similar response times for the tasks, except for the task 9 × 15, where the patients spent slightly, but not significantly longer time (*t* = 2.42, Bonferroni‐corrected *p *= .126) (Figure 2).

### 3.4 Pupillary responses to problem‐solving

**Figure 3:**
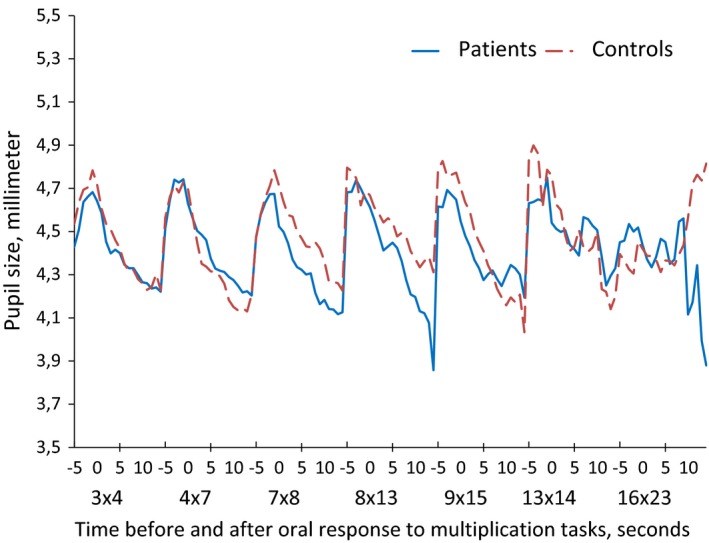
Pupil size of patients and controls during mathematical tasks of increasing difficulty. The patients and controls had similar curves of pupillary responses to mathematical tasks. Both groups showed a pupillary dilation with a maximum in the time interval from 1 s before and to 1 s after the oral response to the mathematical tasks

*Initiation time* was defined as the time when a new auditory task was given, and oral response was defined as the time when the research assistant registered the response from the participant (Figures 1 and 3).

The *maximum pupil size* of patients and controls was observed in the time interval from 2 s before the oral response to 1 second after the response, as illustrated in Figure 1. We therefore defined the *task‐related pupillary dilation* as the difference between the maximum pupil size in this time interval and the pupil size at rest before each task. This measure of pupillary dilation, in millimeters, was used in the further data analyses.

Patients and controls had similar curves of pupillary dilation during problem‐solving, as illustrated in Figure 3.

The term *task accumulated pupil (TAP) size* was used to describe the pupil size at baseline before each new experimental task.

The patients and controls had similar pupil size at baseline, and both patients and controls had increasing TAP size as the experiment advanced (ANOVA: interaction effect: Wilks lambda = 0.92, *F*_6,76_ = 0.14, *p *= .328, main effect: Wilks lambda 0.512, *F*_6,76_, *p *< .001, partial eta squared = 0.488, between‐groups effect: *F*_1,81_ = 0.14, *p *= .709).

We collapsed the pupillary dilation for all tasks and performed an ANCOVA with age, gender and baseline pupil size as covariates, group (patient or control) as a fixed factor and pupillary dilation as dependent variable (Table 3). There was no difference in pupillary dilation in response to the mathematical tasks between the groups (*F *= 1.31, partial eta square = 0.063, *p *= .272). We further tested for differences in pupillary dilation between the groups for each of the different mathematical task in order to see whether the groups would behave differently with increasing task difficulty. There were no group differences in pupil dilation with increasing task difficulty (data not shown).

**Table 3:** Pupillary dilation during response to mathematical tasks

	Patients *n *= 41	Controls *n *= 42	ANCOVA		
	Pupillary dilation, mm (*SD*)	Pupillary dilation, mm (*SD*)	*F*	Partial eta square	*p*‐value
Pupillary dilation, all tasks	0.55 (0.26)	0.54 (0.29)	1.31	0.063	.272

### 3.5 Pupillary responses of different subgroups

Both patients and controls generally performed well on the neuropsychological tests. Regression analyses showed that the scores for processing speed (SDMT), were not associated with the pupillary dilations of the participants in general (*r *= −.002, *p *= .985), nor in the MS patient group alone (*r *= −.075, *p *= .690). Further, we found no correlation between verbal memory (CVLT) and pupillary dilation, neither in the whole group (*r *= 0.060, *p *= .599) nor in the patients (*r *= −.007, *p *= .966). There was also no correlation between performance on the visuospatial tests and pupillary dilations in all participants (*r *= 0.171, *p *= .131), nor in the patients (*r *= 0.261, *p *= .108). Controlling for age, gender, and baseline pupil size did not alter these results (data not shown).

**Figure 4:**
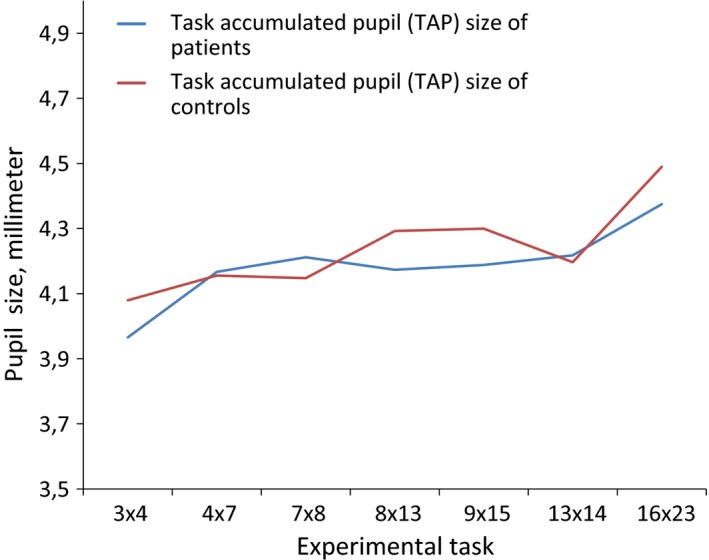
Task accumulated pupil (TAP) size of patients and controls before each new task. Both patients and controls had increasing TAP size as the experiment advanced

**Figure 5:**
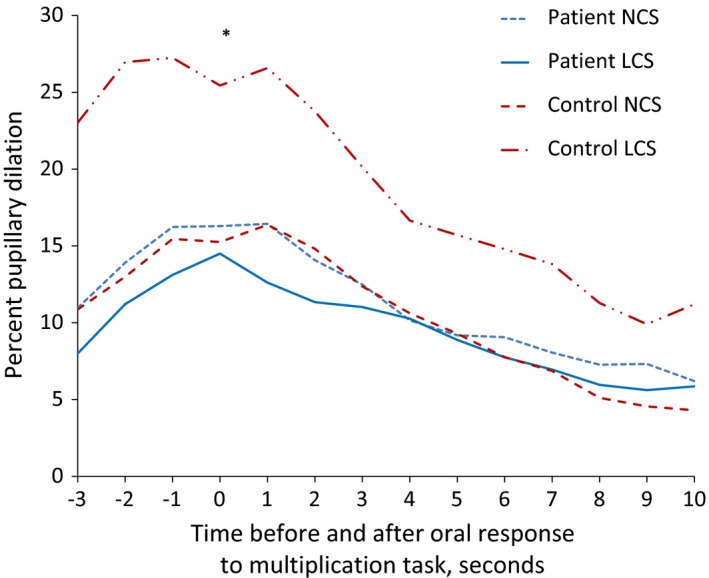
Pupillary responses of different subgroups. The mean of the correct responses of the three easiest tasks is shown for different subgroups of patients and controls. In (a) the LCS controls have a significantly larger pupillary response than the NCS controls. In (b) there are no significant differences between the patients with or without fatigue and in (c) there are no significant differences between patients with or without depressive symptoms. LCS: low cognitive score, NCS: normal cognitive score, *Significant difference in response between patients and controls with LCS, *p *< .05

However, in a subgroup of both patients (*n *= 9) and controls (*n *= 6) classified as LCS (low cognitive score) the pupillary response was significantly larger than both NCS controls and patients (Figure 4). The LCS patients had significantly smaller pupillary responses than the LCS controls (*F*_2,25_ = 8.10, *p *= .009, Wilks lambda = 0.131, partial eta squared: 0.245). There was no significant difference in pupillary response between LCS and NCS patients (*F*_13,18_  = 0.375, *p *= .961, Wilks lambda = 0.787) (Figure 5).

**Figure 6:**
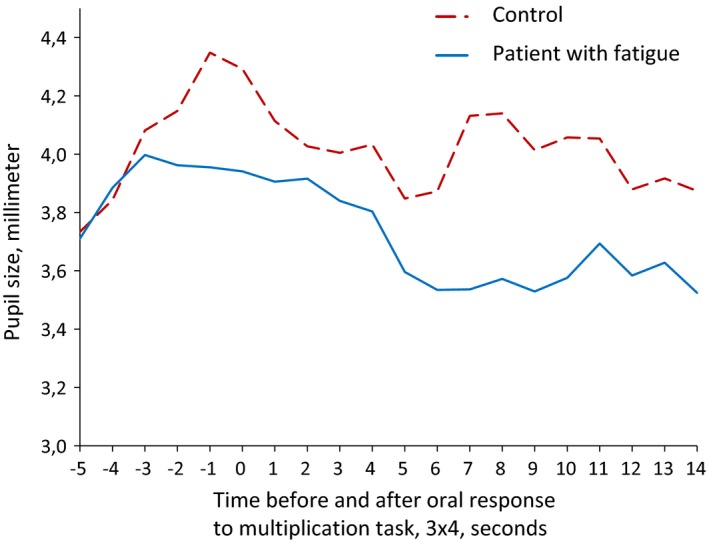
Patients with and without fatigue

Patients were divided into subgroups with no fatigue (FSS≤4, *n *= 21) and with fatigue (FSS >4, *n *= 19). There was a tendency toward smaller pupillary responses in patients with fatigue compared to the non‐fatigue group (*F*_2,30_ = 2.60, *p *= .118, Wilks lambda = 0.150). When examining the curves of pupillary dilation of healthy controls and patients with and without fatigue, we noted that some of the patients with fatigue seemed to have a different profile of the pupillary response to problem‐solving compared to healthy controls and non‐fatigued patients. An example is given for the simple task for a typical healthy patient and a fatigued patient (Figure 6).

As illustrated, the fatigued patient has a smaller pupillary response, and the response lasted for slightly longer than the normal pupillary response.

The patients were further divided into groups of no depression (BDI≤12, *n *= 30) and depression (BDI >12, *n *= 10). We found no significant association between depression scores and pupillary responses (*r *= −.251, *p *= .119).

### 3.6 The effect of possible confounders on the pupillary responses

Approximately, half of the patients had a history of optic neuritis. This could interfere with the pupillary response, and we therefore tested whether pupillary dilations were different between patients with and without a history of optic neuritis on the non‐tested eye. To test this, we performed an ANCOVA with pupillary dilations for all the mathematical tasks as the dependent variable and a history of optic neuritis as the fixed factor. We found no differences between the groups (*F *= 1.056, partial eta squared = 0.026, *p *= .310). We further controlled for age, gender, and baseline pupil size with similar results (data not shown). The patients’ pupil size at baseline was unrelated to RNFL (*r *= −.019, *p *= .908) and VEP (*r *= 0.171, *p *= .292). Moreover, the peak of pupillary dilation was unrelated to RNFL (*r *= −.09, *p *= .593) and VEP (*r *= −.257, *p *= .109).

Sixty‐nine percent of the patients had visible brainstem lesions on MRI. There was no difference in the pupillary response of the patients with and without brainstem lesions (*t* = 0.668, *p *= .509).

## 4 DISCUSSION

In this study, we investigated the pupillary response to problem‐solving in early MS patients and healthy controls. Both patients and controls showed similar pupillary responses to cognitive tasks at a group level. However, while LCS controls had an increased pupillary response to cognitive tasks, LCS MS patients did not show such an increased response. We further identified a nonsignificant altered pupillary response in a subgroup of fatigued patients. Thus, we conclude that the early MS patients as a group had similar task‐evoked pupillary responses as their healthy controls. This indicates that for most of the patients task‐related allocation of attention is well‐functioning, facilitating efficient cognitive processing. This is in accordance with the recent fMRI results of cognitive processing of early MS patients, where normal activation patterns were found in the cognitively preserved patients (Rocca, Valsasina, et al., 2014).

Our patient group succeeded as well as the controls on both the neuropsychological tests and the mathematical tasks, indicating that they had no obvious cognitive difficulties in the domains of processing speed, short‐term memory, and problem‐solving. We have previously reported that the patients mostly were in part‐ or full‐time work (Nygaard et al., 2015), and their high level of education also underscore their status as a well‐functioning group. The “cognitive reserve” hypothesis in MS has suggested that cognitive decline can be delayed in patients with high education or intellectual leisure habits, even in the presence of structural brain changes (Sumowski et al., 2013). In line with this, we have recently published results on cognition and gray matter changes from the same cohort showing that the patients performed well on all domains of cognition tested, in spite of widespread gray matter thinning (Nygaard et al., 2015).

We identified different pupillary responses between LCS controls and LCS patients. As expected, the LCS controls had larger task‐related pupillary responses to the easy mathematical task, which indicated that they experienced a higher processing load than the NCS controls. The LCS patients lacked this extra recruitment. On the contrary, the LCS patients had a tendency toward a smaller task‐related pupillary response than the NCS patients. We propose that this reduced response to cognitive tasks may index a neural deficit that can contribute to a worse cognitive performance.

Although the patients and controls had similar task‐related pupillary responses on a group level, we observed that some of the fatigued patients had different response curves. These patients appeared to have a reduced and more prolonged pupillary response to problem‐solving than healthy controls. We propose that this reduced response to the cognitive tasks may index a neural deficit, likely affecting connectivity of brain areas to the norepinephrine‐Locus Coeruleus system that will in turn be expressed in the pupil and contribute to a worse cognitive performance.

Interestingly, studies of patients with Alzheimer's or Parkinson's disease have shown an association between a slower pupillary light reflex and cognitive impairment, indicating that a cholinergic deficiency (affecting the parasympathetic branch of oculomotor control on the pupillary muscles) may be related to both the cognitive and the autonomic alterations in these patient groups (Fotiou et al., 2009). Thus, a central autonomic dysregulation in neurological diseases may be associated with cognitive dysfunction.

The smaller pupillary response in some fatigued patients observed in this study suggest central tiredness as the causal link between fatigue and pupillary responses. Lowenstein and Loewenfeld proposed a model of disintegration of central control due to post‐task fatigue expressed as a change in the shape of the pupillary light reflex curve (Lowenstein & Loewenfeld, 1951), and it is also known that sleep deprivation changes the pupillary light reflex curves (Wilhelm, Wilhelm, Lüdtke, Streicher, & Adler, 1998). As fatigue and sleep disorders are tightly linked in MS patients (Veauthier et al., 2011), the altered pupillary response in our subgroup of fatigued MS patients may be related to a central tiredness caused by changes in Locus Coeruleus projections to sleep‐promotion nuclei (Szabadi, 2013). Both patients and controls were randomly examined throughout the working hours; therefore, the effect of sleepiness in both groups should be evenly distributed. The baseline measures before the first cognitive task shows no difference between the groups and suggests that we have corrected for different levels of wakefulness. In normal sleepiness, we would have expected to find a reduced pupillary diameter at baseline if one of the groups was significantly more sleepy than the other (Wilhelm et al., 1998). Our results may therefore touch upon the true nature of fatigue as a condition different from sleepiness. This could in part explain why testing of autonomic instability by pupillary unrest (PUI) measurements done by Egg et al. did not show an association with MS‐related fatigue, since PUI is a test of arousal and sleepiness (Egg, Högl, Glatzl, Beer, & Berger, 2002). Studies of the association between fatigue, cognition, and autonomic dysfunction in MS have shown conflicting results and warrants further research (Flachenecker, 2007; Flachenecker et al., 2003; Keselbrener et al., 2000).

While fMRI identifies altered recruitment of neural networks, the pupillometry results presented here could indicate a central autonomic dysregulation caused by reduced activity in the cognitive alert system of MS patients. Both an ongoing inflammatory process in MS patients and a more disseminated disease could lead to a decreased autonomic response, expressed as reduced pupillary dilation in response to cognitive tasks (Wilhelm & Wilhelm, 2003). This is in line with other studies of the pupillary light reflex in early MS patients, that have identified both sympathetic and parasympathetic disturbances without associations to neurological disability (Pozzessere et al., 1997; Surakka et al., 2008).

Reduction in CNS's noradrenergic levels and damage to Locus Coeruleus neurons have been described both in the experimental autoimmune encephalitis (murine MS model) and in autopsies of chronic MS patients, (Polak, Kalinin, & Feinstein, 2011). The same group has found that Locus Coeruleus damage increases the symptom severity in experimental autoimmune encephalitis, and that increasing noradrenergic and noradrenergic precursor levels can reverse this effect (Simonini et al., 2010). Treatment of experimental autoimmune encephalitis mice with a vincamine derivate vindeburnol, which temporarily depletes CNS noradrenergic and promotes an upregulation of Locus Coeruleus's noradrenergic levels and metabolism, leads to a reduction in experimental autoimmune encephalitis symptoms (Polak et al., 2012). A randomized controlled trial of treatment to normalize noradrenergic levels of the CNS (lofepramine, phenylalanine, and B12) in 69 MS patients in different stages of the disease lead to a reduction in MS symptoms in the treated patients (Wade, Young, Chaudhuri, & Davidson, 2002) and, in a small subgroup of patients, to a reduction in hypointense T1 lesions and a slower atrophy rate on MRI (Puri et al., 2001). Thus, there are indicators, though limited in number and based on small and mixed patient samples, that Locus Coeruleus's noradrenergic levels are reduced and that a reversal of this reduction may improve symptoms in both animal models and MS patients. This Locus Coeruleus ‐noradrenergic pathology may explain the small task‐related pupillary response of some patients in this study.

**Figure 7:**
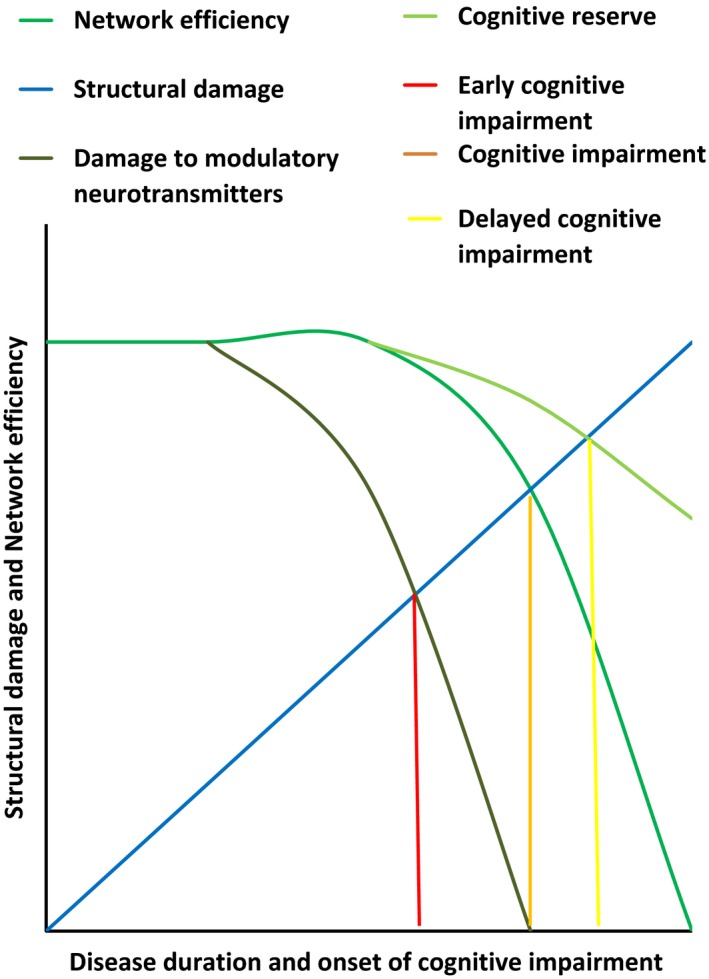
Factors contributing to the onset of cognitive impairment in MS patients. Probably both structural damage and network collapse contributes to the onset of cognitive impairment in MS patients. Damage to the regulation of strategic modulatory neurotransmitters may expedite cognitive impairment, while cognitive reserve may delay such a collapse

A recent opinion paper has proposed that cognitive impairment in MS occurs as a result of network collapse (Schoonheim, Meijer, & Geurts, 2015). We propose that such network collapse could be expedited by a damage to the regulation of modulatory neurotransmitters. Cognitive reserve, on the other hand, may delay such a collapse. We therefore expand Schoonheim and colleagues’ model of cognitive impairment as illustrated in Figure 7.

Our study has several limitations. First, these are the baseline results of a cohort study. We do not have longitudinal data and cannot draw conclusions concerning causality. Surprisingly, the patient group was cognitively well‐functioning and we therefore could not study a cognitively impaired patient group. Studies of patients with a longer disease duration and a larger proportion of patients with low cognitive scores should be performed to assess whether a reduced Locus Coeruleus's activation may be linked to cognitive impairment, depressive symptoms, and fatigue in MS patients. Further, MS pathology may cause both focal and global CNS damage. We have as far as possible corrected for confounding factors by performing careful ophthalmological examinations, age and gender matching of patients and controls, similar timing of the examinations, and identical test situations, but we cannot distinguish between a local Locus Coeruleus's dysfunction and other CNS changes as a cause. Medications such as antidepressants could have interfered with autonomic function. Our participants were asked for a full list of currently used medications and only one patient was on antidepressive treatment (amitriptylin hydrochlorid). In this study, we did not test for clinical symptoms of dysautonomia which would have added further insight into these complex possible neurological associations. This warrants further studies including test for differences in patients with low versus normal cognitive score and fatigued versus non‐fatigued differ in terms of autonomic function.

The experiment consisted of mathematical tasks of increasing difficulty and some of the participants may have been particularly stressed by the nature of the tasks, possibly leading us to measure a stress response instead of just cognitive load. With our study design, we cannot distinguish between the autonomic response caused by stress as in fear of not mastering the task, and by alertness caused by the mental load applied to perform the actual task. A normal stress response depends on a state of alertness. Studies of the association between dysfunction of alertness and fatigue have found that these two phenomena are closely correlated in MS patients (Weinges‐Evers et al., 2010). Indeed deficits in alertness networks have been found in MS patients and could explain some aspects of cognitive dysfunction in these patients (Urbanek et al., 2010). Thus, a possible difference in autonomic response between patients and controls could be caused by dysfunctional alertness networks leading to a different stress response in the patients. Another limitation is the fact that the healthy controls were not tested for fatigue and depression. This would have contributed to clarify the impact on cognitive performance in healthy individuals and it would have been an additional strength to our analyses. We did not interview our participants about sleep problems and the distinction between fatigue and sleepiness/alertness is difficult to draw only based on FSS and baseline pupil size, this must be taken into account when drawing conclusion about these results. Due to the design of the experiment, it is difficult to evaluate the effect of the hard tasks, as the error rate was very high. Future studies should therefore attempt at a design in which the effect of the increasing task difficulty would be easier to monitor.

Our relatively limited number of participants in the subgroup analyses constitutes another limitation of our study and warrant further replication in a larger sample size to prove the usefulness of pupillary responses as a reliable proxy to test levels of central neuropeptides.

In conclusion, this may be the first study to investigate pupillary responses to cognitive tasks in MS patients. The pupillary responses were similar comparing patients and controls. We propose that a reduced response to cognitive tasks may index a neural deficit that can contribute to a worse cognitive performance. Further, some fatigued patients did not show the same activation as the healthy controls, indicating that the Locus Coeruleus's response in MS patients with symptoms of fatigue may be altered.

Pupillometry research in psychiatric patients has had a sharp increase in recent years, mainly due to the reduced costs and the easy administration compared to MRI. Accordingly, the knowledge about pupillometry in patients with psychiatric diseases has accumulated (Graur & Siegle, 2013). With basis in our evidence that task‐related pupillary responses are preserved in patients with a history of ON or brainstem lesions and preserved vision, we argue that this method could be useful in future studies of cognition, fatigue, and eventually psychiatric symptoms in MS patients longitudinally from early on in the disease course and preferably in combination with autonomic testing and fMRI.

Finally, we propose pupillometry as a valid method for unveiling task‐related changes of attention that may link cognition and fatigue in multiple sclerosis patients.

## CONFLICT OF INTEREST

None declared.
